# Bolstering the Business Case for Adoption of Shared Decision-Making Systems in Primary Care: Randomized Controlled Trial

**DOI:** 10.2196/32666

**Published:** 2022-10-06

**Authors:** JoAnn M Sperl-Hillen, Jeffrey P Anderson, Karen L Margolis, Rebecca C Rossom, Kristen M Kopski, Beth M Averbeck, Jeanine A Rosner, Heidi L Ekstrom, Steven P Dehmer, Patrick J O’Connor

**Affiliations:** 1 HealthPartners Institute Bloomington, MN United States; 2 Research Department HealthPartners Center for Chronic Care Innovation Bloomington, MN United States; 3 Genesis Research Hoboken, NJ United States; 4 Medica Health Plan Minneapolis, MN United States; 5 HealthPartners Bloomington, MN United States

**Keywords:** clinical decision support, primary care, ICD-10 diagnostic coding, CPT levels of service, shared decision-making

## Abstract

**Background:**

Limited budgets may often constrain the ability of health care delivery systems to adopt shared decision-making (SDM) systems designed to improve clinical encounters with patients and quality of care.

**Objective:**

This study aimed to assess the impact of an SDM system shown to improve diabetes and cardiovascular patient outcomes on factors affecting revenue generation in primary care clinics.

**Methods:**

As part of a large multisite clinic randomized controlled trial (RCT), we explored the differences in 1 care system between clinics randomized to use an SDM intervention (n=8) versus control clinics (n=9) regarding the (1) likelihood of diagnostic coding for cardiometabolic conditions using the 10th Revision of the International Classification of Diseases (ICD-10) and (2) current procedural terminology (CPT) billing codes.

**Results:**

At all 24,138 encounters with care gaps targeted by the SDM system, the proportion assigned high-complexity CPT codes for level of service 5 was significantly higher at the intervention clinics (6.1%) compared to that in the control clinics (2.9%), with *P*<.001 and adjusted odds ratio (OR) 1.64 (95% CI 1.02-2.61). This was consistently observed across the following specific care gaps: diabetes with glycated hemoglobin A_1c_ (HbA_1c_)>8% (n=8463), 7.2% vs 3.4%, *P*<.001, and adjusted OR 1.93 (95% CI 1.01-3.67); blood pressure above goal (n=8515), 6.5% vs 3.7%, *P*<.001, and adjusted OR 1.42 (95% CI 0.72-2.79); suboptimal statin management (n=17,765), 5.8% vs 3%, *P*<.001, and adjusted OR 1.41 (95% CI 0.76-2.61); tobacco dependency (n=7449), 7.5% vs. 3.4%, *P*<.001, and adjusted OR 2.14 (95% CI 1.31-3.51); BMI >30 kg/m^2^ (n=19,838), 6.2% vs 2.9%, *P*<.001, and adjusted OR 1.45 (95% CI 0.75-2.8). Compared to control clinics, intervention clinics assigned ICD-10 diagnosis codes more often for observed cardiometabolic conditions with care gaps, although the difference did not reach statistical significance.

**Conclusions:**

In this randomized study, use of a clinically effective SDM system at encounters with care gaps significantly increased the proportion of encounters assigned high-complexity (level 5) CPT codes, and it was associated with a nonsignificant increase in assigning ICD-10 codes for observed cardiometabolic conditions.

**Trial Registration:**

ClinicalTrials.gov NCT 02451670; https://clinicaltrials.gov/ct2/show/NCT 02451670

## Introduction

Care delivery systems are increasingly considering an array of software products that promote clinical decision support (CDS), care efficiency, and shared decision-making (SDM) in primary care environments. CDS uses computable biomedical information, person-specific data, and inferencing mechanisms to generate helpful information to clinicians, patients, and care teams, as care is being delivered with the objective of reducing errors and adverse events and promoting best practices [1]. CDS can also be used to generate SDM interfaces to facilitate patient engagement and help patients make choices, incorporate personal preferences, and help them prioritize clinical recommendations and decisions [2]. Key features shown to improve the success of SDM products include incorporating them into clinician workflows without disruption, delivery at the right time in the clinical encounter to influence decision-making, and provision of SDM output to patients as well as clinicians [3]. We developed an SDM system that involves patient-centered CDS and a workflow that presents clinicians and patients with printed information about chronic care gaps in low- and high-literacy formats and prioritizes care options based on potential benefits to the individual early in primary care encounters. We have shown that an SDM system with these features achieves high clinician satisfaction rates and sustainable high SDM use, improves glucose and blood pressure (BP) control in patients with diabetes mellitus (DM), lowers 10-year cardiovascular (CV) risk in patients without DM or heart disease, and positively influences the frequency and quality of SDM [4-6].

However, many care systems are operating with tight budgets and facing difficult choices regarding adoption of SDM due to the cost of implementation and maintenance of SDM technology [3,7]. Very few studies have assessed the cost-effectiveness of SDM, and those that have invariably adopt the societal or health insurer perspective. In 1 such study, Gilmer et al [8] estimated the base-case incremental cost-effectiveness of implementing a clinical decision- making system used in SDM for patients with DM at US $3017 per quality adjusted life year gained [8]. This amount was considered cost-effective by usual standards from a payer perspective [9]. However, most of the cost burden for implementing SDM falls on the care delivery system rather than the payer, and the lack of data needed to estimate the impact of SDM implementation on care delivery system revenue is often cited as a major barrier to adoption [7,10].

The objective of this analysis was to evaluate the impact of an SDM system on diagnostic coding and billing at primary care encounters because these factors substantially impact revenue generation for a care delivery system and can ultimately influence the case for SDM adoption. In a care delivery model that relies on “fee for service (FFS)” reimbursement, it is important that care systems are able to capture billing codes that reflect the extent to which SDM might increase the amount of time, number of clinical issues addressed, and complexity of medical decision-making at patient encounters [11]. In today’s emerging transition to value-based care agreements, accurate and complete diagnostic coding is related to risk-adjusted reimbursement for the population served. In most health care settings today, both adequate billing service levels and accurate coding of conditions are necessary to optimize revenue and optimally manage the health care needs of the patients and populations they serve [11].

The SDM system studied in this analysis included no specific components to encourage diagnostic coding or influence billing codes. However, it directed clinician attention to care gaps related to diabetes and uncontrolled CV risk factors, and it would be expected to indirectly influence diagnostic coding and billing. Therefore, in an exploratory analysis conducted as part of a multisite randomized controlled trial (RCT) to evaluate the quality impact of the SDM system on patients with high CV risk and serious mental illness, we assessed the effect of using SDM at intervention clinics within 1 medical group on (1) rates of diagnostic coding for SDM-related clinical domains based on the 10th Revision of the International Statistical Classification of Diseases and Related Health Problems (ICD-10) [12] and (2) current procedural terminology (CPT) codes in the level of service used for billing at clinics using the evidence-based SDM system [13].

## Methods

### Study Design and Study Population

This analysis occurred as part of a larger multisite clinical trial (trial registration: NCT 02451670) funded by the National Institute of Mental Health that developed, implemented, and evaluated an SDM system for adults with serious mental illness (SMI), such as schizophrenia, schizoaffective disorder, or bipolar disorder, who die on average 17 years earlier than the rest of their birth cohort, primarily due to CV disease [14]. The objective of the study was to determine if an SDM system targeting reversible CV risk factors would lower the reversible 10-year CV risk for patients with SMI over 12-18 months. The study showed that the rate of increase in the total modifiable CV risk was 4% lower among intervention patients compared to the control, emphasizing the value of using the SDM system for the prompt management of modifiable CV risk factors in the SMI population [15]. Of the 3 participating medical groups, 1 was used for this exploratory analysis of SDM impact on billing and coding. The SDM implementation at this site also included patients with diabetes, CV disease, and high CV risk in addition to those with SMI. For this analysis, we explored the impact of the SDM system on diagnosis and CPT coding at all encounters of adult patients with diabetes, SMI, CV disease, or high reversible CV risk, plus suboptimal control of 1 or more major CV risk factors. The specific inclusion and exclusion criteria are described in more detail below. In this medical group, 17 primary care clinics were randomly assigned to receive (n=8) or not receive (n=9) the SDM system beginning March 15, 2017. The control clinics were scheduled to receive the SDM system 18 months later, in September 2018. The clinic randomization was conducted using a computer-generated random allocation sequence while ensuring a balance in terms of the clinic size and percentage of patients with Medicaid insurance. Clinic names were concealed until intervention assignment.

Inclusion and exclusion criteria for study analysis eligibility were determined for each study-eligible patient by SDM algorithms at the start of every primary care encounter and included the following.

#### Inclusion Criteria

These criteria include an office encounter in a primary care department with a patient aged 18 to 75 years and one of the following two clinical criteria:

1. The presence of DM, CV disease, or SMI and not meeting evidence-based goals for one or more of the following major CV risk factors were considered: statin use [16], BP [17], glycemic control [18], weight (BMI>25 kg/m^2^) [19], tobacco cessation [20], and aspirin use, if indicated [21,22].

2. The reversible 10-year CV risk score was greater than 10% (without DM, CVD, or SMI identified). The reversible CV risk score was the sum of the amount of 10-year CV risk attributable to each of the above risk factors that could potentially be eliminated if the patient were to achieve the guideline-recommended clinical goal. For weight, the reversible risk was the amount of reversible CV risk attributable to a drop of 3 units in the BMI (kg/m^2^), which is equivalent to a 10- to 20-pound weight loss for most individuals.

#### Exclusion Criteria

ICD-10 visit codes and problem list codes were used to exclude all patients with one or more of the following conditions: hospice or nursing home, active cancer, current or recent pregnancy, and cognitive impairment.

### Intervention Description

The evidence-based SDM system directs patient and clinician attention to a patient’s personalized care priorities at the point of care. A custom routine programmed in the electronic health record (EHR) gathers key clinical data and securely exchanges it with a web service, where algorithms are applied to identify and prioritize evidence-based CV risk factor care improvement options for patients and clinicians. The algorithms use published risk-prediction equations [23-25] to calculate an individual’s reversible CV risk potential and then prioritize out-of-control CV risk factors in a list form from the most to the least likely to lower CV risk if successfully addressed. Care suggestions include personalized pharmacologic and lifestyle treatment options that account for the patient’s current therapy, most recent status of clinical values, distance from clinical goals, and relevant comorbidities. The SDM also provides safety alerts (eg, for drug contraindications and interactions), screening and monitoring reminders, and suggestions for appropriate follow-up intervals [5].

The SDM system is automatically triggered at adult visits when clinic rooming staff enter any BP value into the EHR, as is the case in over 95% of all primary care clinic visits. When web-based clinical algorithms identify a patient who meets the study eligibility criteria, a flag is returned to the EHR that triggers an EHR best practice advisory (BPA) pop-up inviting clinic rooming staff to open (1 click) and print (1 click) the SDM tools for patients and clinicians in intervention clinics. Having paper interfaces available to clinicians and patients at the beginning of the encounter was key to the SDM process and workflow, and using rooming staff to print the interfaces was key to ensure high use rates and exposure to the SDM tools.

### Printed SDM Interfaces for Patients and Clinicians

**Figure 1:**
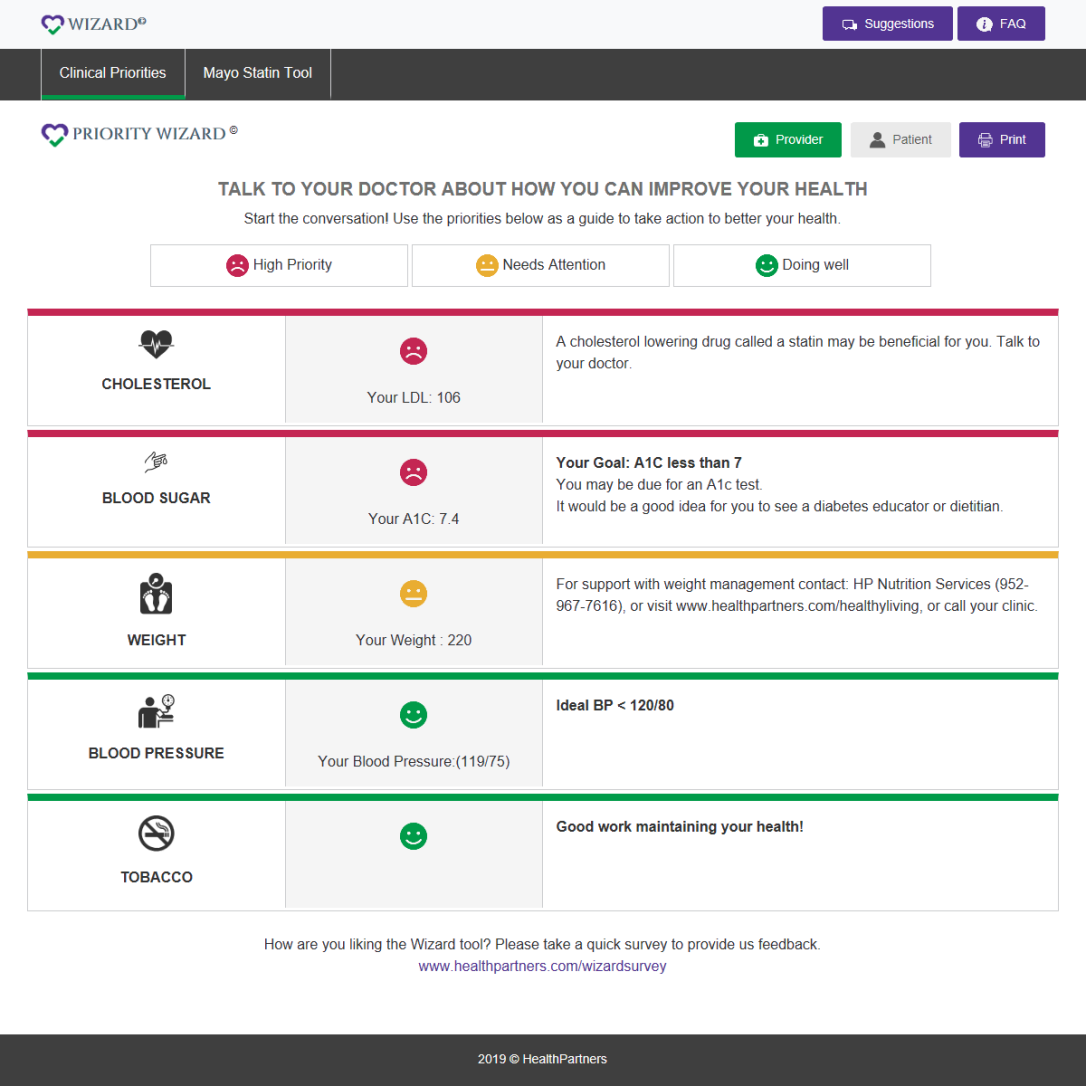
Example of the printed shared decision-making interfaces for patients. A_1c_: glycated hemoglobin; BP: blood pressure; LDL: low-density lipoprotein.

To meet a wide range of health literacy needs, the printed SDM tools included a more detailed “clinician-oriented” decision support interface as well as a companion low-literacy “patient-oriented” interface. The interfaces went through multiple iterations based on feedback received from clinicians and patients during the study. Figure 1 shows the version of the SDM system for a synthetic patient.

The lay/patient version is printed by the rooming nurse and given to the patient to review while waiting in the exam room for the provider, with the following message: “*If you act on the things with high priority or needs attention, you may be able to reduce your danger of a stroke or heart attack. Talk to your doctor about things you can do.*”

**Figure 2:**
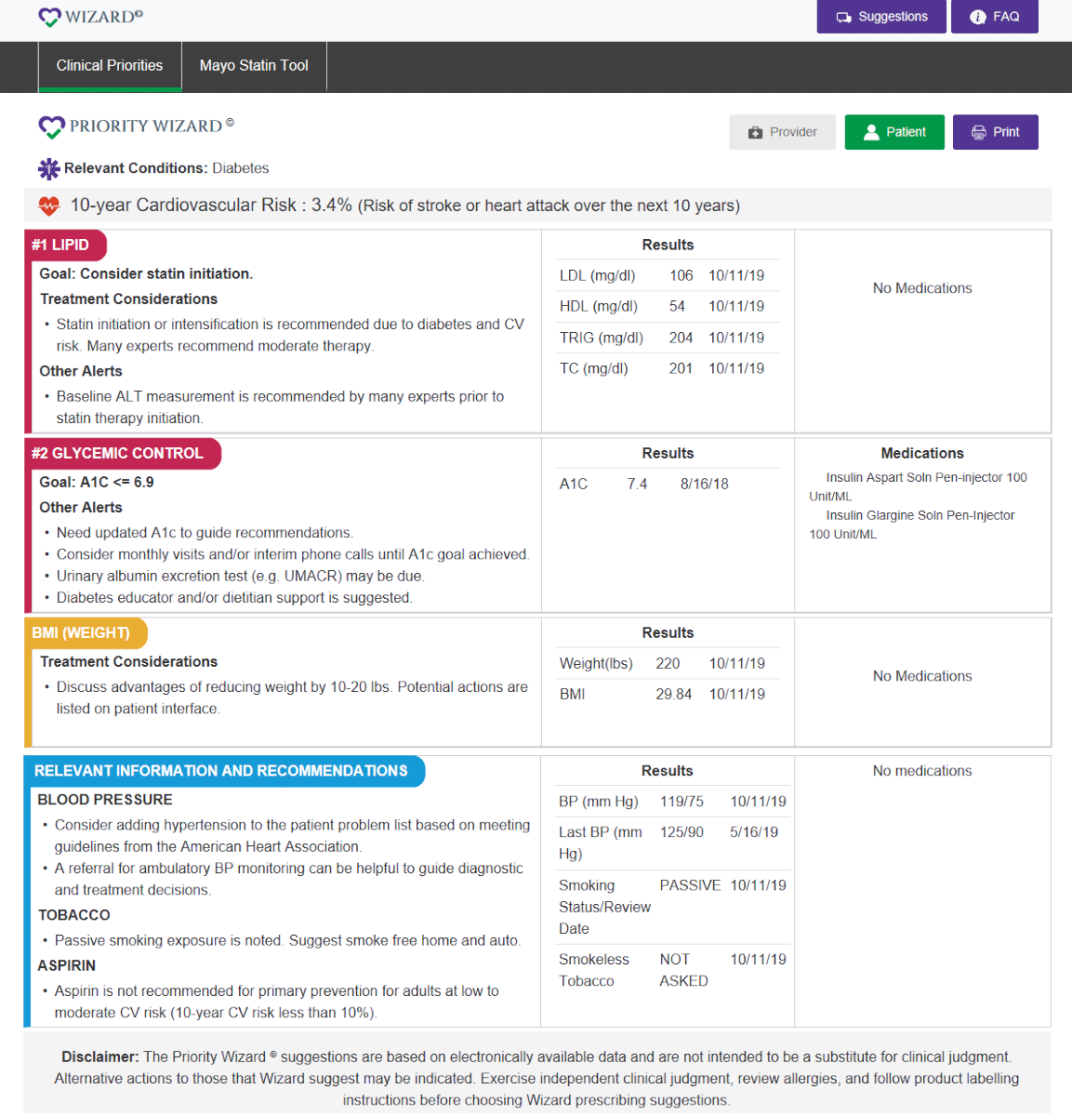
Example of the printed shared decision-making interfaces for clinicians. A_1c_: glycated hemoglobin; ALT: alanine amino-transferase; BP: blood pressure; CV: cardiovascular; HDL: high-density lipoprotein; LDL: low-density lipoprotein; TC: total cholestrol; TRIG: triglycerides; UMACR: urine microalbumin to creatinine ratio.

The professional/clinician version in Figure 2 is printed by the rooming nurse and placed on the exam room door for rapid review by the provider just before the visit. Uncontrolled CV risk factors are prioritized by the potential absolute risk reduction that may be achieved by managing those risk factors. The data presented in Figure 2 are obtained from web service interfaces for synthetic patients and are not from actual patients.

### Recommended Workflow

**Figure 3:**
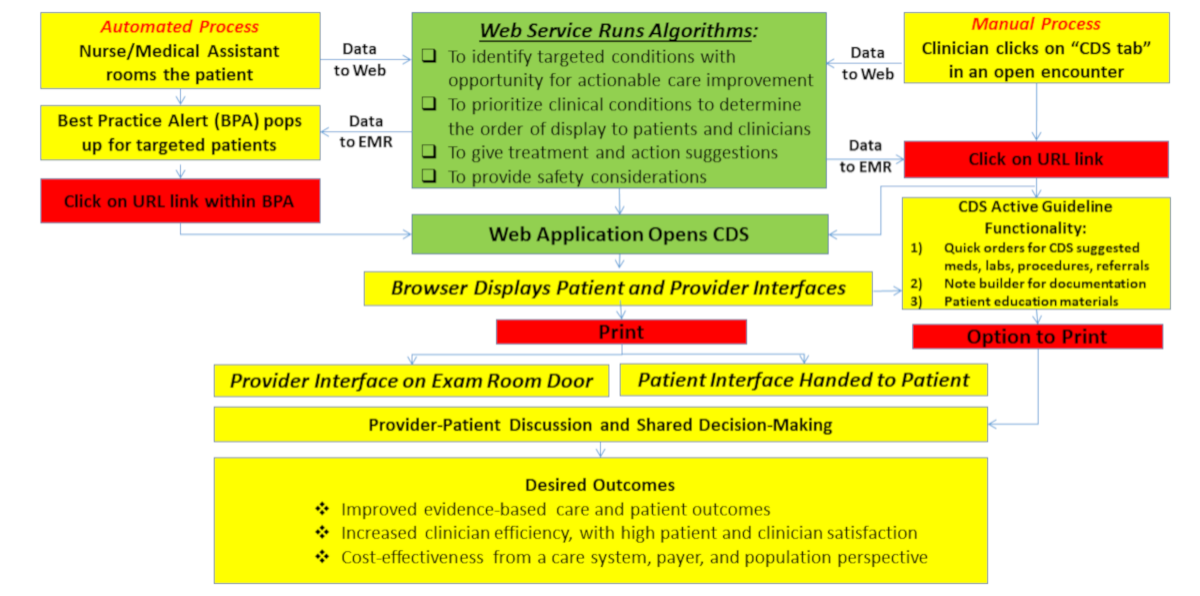
Workflow for shared decision-making use in primary care encounters. CDS: clinical decision support; EMR: electronic medical record.

The automated BPA pop-up for targeted patient encounters in the recommended workflow shown in Figure 3 was for rooming staff to print and give the patient-oriented interface to patients while they waited for the clinician, a design that promoted engagement and improved efficiency when making important decisions for care priorities. The “clinician-oriented” interface was given to clinicians before the encounter to review patient CV risk factor–related clinical priorities. Clinicians at intervention clinics also could manually view the SDM within the EHR for any adult patient (independent of study eligibility or CV risk) from an SDM activity tab visible in all open encounters. Later in the study, when the SDM was opened from this tab, the SDM display included active guideline features that facilitated quick orders for medications, labs, procedures, and referrals based on recommendation options generated by the SDM algorithms. Rooming staff in the control clinics did not receive the BPA and clinicians could not display the SDM tools. Providers in both intervention and control clinics could use a “smart dot phrase” within encounter notes to summarize and document the patient’s 10-year CV risk score and CV risk factors not at goal.

### Technical Description of the SDM System Functionality

**Figure 4:**
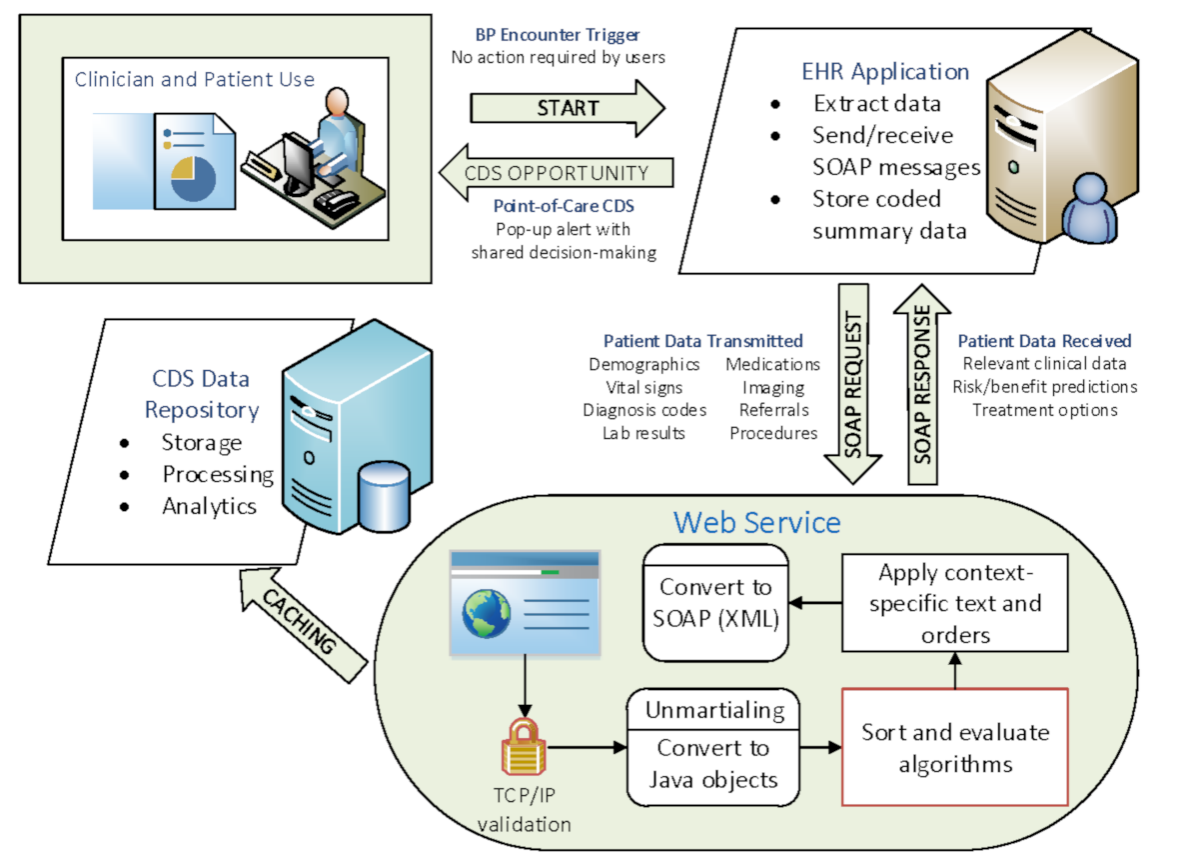
Technology behind the shared decision-making system. BP: blood pressure; CDS: clinical decision support; EHR: electronic health record; SOAP: Simple Object Access Protocol; TCP: Transmission Control Protocol.

The SDM system shown in Figure 4 consists of three interconnected components: (1) a custom routine to extract data from the EHR, (2) web services running on server clusters that process algorithms, and (3) a website that displays the SDM patient and clinician interfaces. The first component of the SDM system involves installation of a program using a Massachusetts General Hospital Utility Programming System (MUMPS) routine in Epic’s database management system (Epic Systems Corporation) called Chronicles. When the BP is entered at the encounter, it triggers the MUMPS routine to extract all the data needed to run the algorithms and packages the information into a Simple Object Access Protocol messaging request that uses text in the XML format. Epic’s Interconnect Infrastructure is used to connect to the web service over https that contains a unique web service call identifier. The web service then processes the data, runs algorithms, stores the unique call identifier, and returns results to the EHR. The EHR code then processes the response and extracts and saves relevant pieces of information into discrete data fields. For targeted patients with care gaps identified within the web service response, the BPA contains a URL link to the web application that displays the SDM tools. When the rooming staff or clinician clicks on the URL link containing the unique patient identifier, the patient’s personalized SDM tools are displayed in real time within the EHR browser. With 1 additional click, the tools can also be printed for patients and clinicians to use in SDM. To the end user, the process to display the SDM takes less than 2 seconds and appears to be entirely integrated within the EHR experience. All data are exchanged via transport layer security with extra layers of security enforced via exchange of unique identifiers and IP address authorization. The SDM system currently uses Epic web services where possible, and these can be replaced with Fast Healthcare Interoperability Resources (FHIR) as FHIR features mature and offer improved interoperability with other EHR systems and software.

### Training

Intervention clinic staff were offered a 1-hour luncheon training to introduce the SDM tools and learn the recommended workflow before the SDM system became available on March 15, 2017. Over the intervention period, nursing leaders at intervention clinics were given monthly reports of how often the tool was being printed for the target population. From our previous implementation experience, these monthly process measurements were essential for promoting and maintaining high SDM use rates. With this implementation process and workflow, the SDM was printed for 75% of eligible encounters on average after the first month, and these rates were sustained for the duration of the intervention.

### Analysis

On entering the BP for all encounters, data exchange with the SDM web service occurred in both the SDM system intervention and control clinics for analysis purposes and was saved in a data repository, but the SDM tools were displayed only at the SDM system intervention clinics. We evaluated all eligible patient encounters occurring from March 15, 2017, to December 31, 2017, in the intervention and control clinics. Encounter-level data from the SDM repository were later merged with data extracted from the EHR (Epic Clarity) [26], which included the ICD-10 visit diagnostic codes and CPT level of service for the same encounters. The objective of this analysis was to evaluate differences between the intervention and control clinics in terms of the following: (1) likelihood of ICD-10 diagnostic coding for DM (E10-E11), hypertension (I10-I16), hyperlipidemia (E78), obesity (E66), smoking (F17 and Z72), and CVD (G45, I20-25, I63-70, and I74); and (2) CPT billing codes documented by clinicians as straightforward (level 2, CPT 99212), low complexity (level 3, CPT 99213), moderate complexity (level 4, CPT 99214), or high complexity (level 5, CPT 99215).

Documentation of the CPT levels of service was done by clinicians based on the intensity and complexity of medical decision-making at encounters using the recommended criteria related to the nature and number of clinical problems, amount and complexity of the data reviewed, and the risk of morbidity and mortality to the patient [13]. If counseling or coordination of care accounts for more than 50% of the visit, the CPT service level can be based on the length of the visit as well [13].

Descriptive summaries of diagnostic and billing codes were tabulated, including frequencies, means, 95% CIs, and percentiles of the continuous distribution, where applicable. We used the Fisher exact test for unadjusted comparisons between intervention and control clinics. Generalized linear mixed regression models (with a binomial distribution and logit link) were used for covariate adjustment and random intercepts to account for clustering at the provider and clinic levels. All modeling results reported here are adjusted for the age (continuous), gender (female/male), and race (White, Black, and other/unknown) of the patients. The *P* values reported are 2-sided. Analyses were conducted using SAS (version 9.4, SAS Institute) and R (version 3.4.3, R Foundation for Statistical Computing).

### Ethics Approval

The study was reviewed in advance, approved, and monitored by the HealthPartners Institutional Review Board (IRB, reference: 13-154). The IRB approved waiver of written consent from participants.

## Results

### Analysis Population

During the 9.5-month evaluation period, 32,735 primary care encounters with 18,070 unique adult patients were identified. Table 1 summarizes the demographic characteristics of the eligible encounters. Approximately half of the encounters (16,335/32,735, 49.9%) were with female patients; the mean age was 58.1 years (slightly older for the intervention clinics, 59.0 years vs 57.2 years), with 75% (13,553/18,070) of patients being White. The mean BMI was 33.0 kg/m^2^. CVD was present in 20.4% (6678) of the encounters, and of the encounters in which CVD was not identified, the mean 10-year estimated CV risk [27] was 15.8%. Type 2 diabetes was identified in 69.7% (22,816), type 1 diabetes in 4.2% (1375), and hypertension in 67.7% (22,162) of the encounters.

**Table 1:** Demographic characteristics of patient encounters by clinic intervention status, 2017.

Characteristic			All encounters (N=32,735)		Control clinics (N=16,417)		Intervention clinics (N=16,318)	*P* value	
**Age in years, n (%)**									<.001
	18-39	2782 (8.5)		1478 (9)		1289 (7.9)			
	40-49	4386 (13.4)		2529 (15.4)		1877 (11.5)			
	50-59	8642 (26.4)		4515 (27.5)		4145 (25.4)			
	60-69	11,163 (34.1)		5336 (32.5)		5826 (35.7)			
	70-75	5761 (17.6)		2561 (15.6)		3182 (19.5)			
**Gender, n (%)**									.1
	Female	16,335 (49.9)		8110 (49.4)		8208 (50.3)			
	Male	16,400 (50.1)		8307 (50.6)		8110 (49.7)			
**Race, n (%)**									<.001
	White	24,346 (75.2)		12,198 (74.3)		12,418 (76.1)			
	Black	4223 (12.9)		1904 (11.6)		2333 (14.3)			
	Other/unknown	3895 (11.9)		2331 (14.2)		1567 (9.6)			

### Diagnostic Coding

Table 2 shows the proportion of encounters with each condition and care gap identified by the SDM system that included a corresponding visit diagnostic code. There was a significant increase in diagnostic coding for almost all the cardiometabolic conditions that the SDM addressed. Odds ratios (ORs) from generalized linear mixed models were generally consistent with unadjusted comparisons; however, these estimates were not statistically significant, possibly due to a relatively small sample of clinics (N=17).

**Table 2:** Proportion of encounters with the 10th Revision of the International Classification of Diseases coding for clinical conditions and care gaps identified by shared decision-making algorithms based on intervention clinic status, 2017^a^.

Condition subgroup	Control clinics (N=16,417)	Intervention clinics (N=16,318)	*P* value	Adjusted odds ratio	95% CI
Diabetes (N=24,138), n (%)	17,186 (71.2)	18,055 (74.8)	<.001	1.04	0.8-1.35
Diabetes with HbA_1c_^b^ above individualized goal (N=12,786), n (%)	9705 (75.9)	10,280 (80.4)	<.001	1.12	0.86-1.47
Diabetes with HbA_1c_≥8% (N=8463), n (%)	6618 (78.2)	6931 (81.9)	<.001	1.15	0.89-1.49
Hypertension (N=22,127), n (%)	12,834 (58)	13,542 (61.2)	<.001	1.04	0.73-1.48
Hypertension with BP^c^ above goal (N=8515), n (%)	5779 (68.1)	5926 (69.6)	.13	1.04	0.68-1.59
Suboptimal lipid management (N=17,765), n (%)	5330 (30)	5738 (32.3)	.002	1.01	0.67-1.51
ASCVD^d^ (N=6679), n (%)	1656 (24.8)	1803 (27)	.041	1.18	0.93-1.51
Tobacco use (N=7449), n (%)	1952 (26.2)	2451 (32.9)	<.001	1.38	0.98-1.95
BMI≥30 kg/m^2^ (N=19,838), n (%)	2956 (14.9)	3491 (17.6)	<.001	1.19	0.73-1.94

^a^Age (continuous), sex (female/male), and race (White/Black/other) were included in multivariable logistic regression models.

^b^HbA_1c_: glycated hemoglobin A_1c_.

^c^BP: blood pressure.

^d^ASCVD: atherosclerotic cardiovascular disease.

### CPT Levels of Service

Table 3 shows the proportion of encounters coded as “5,” indicating a high-complexity CPT level (as opposed to other lower CPT levels 2-4), for selected subgroups of encounters with targeted care gaps, glycated hemoglobin A_1c_ (HbA_1c_)>8%, hypertension with BP over goal, suboptimal lipid management, obesity, and tobacco dependency. There were higher proportions of encounters with high-complexity codes for all SDM-eligible encounters. This held true for subgroups of encounters with specific identified conditions, including DM, hypertension, statin use not at goal, CVD, smoking, obesity, and high reversible risk (without DM or CVD). In generalized linear mixed models, accounting for clinic clustering and demographic factors, encounters were statistically significantly more likely to be coded as “5” (high complexity) in intervention clinics overall (OR 1.64, 95% CI 1.02-2.61), and in patients with DM and HbA_1c_>8% (OR 1.93, 95% CI 1.01-3.67), and active smokers (OR 2.14, 95% CI 1.31-3.51).

**Table 3:** Proportion of encounters coded as 5, indicating a high-complexity current procedural terminology level for clinical conditions identified by shared decision-making algorithms based on clinic intervention status, 2017^a^.

Condition subgroup	Control clinics (N=16,417)	Intervention clinics (N=16,318)	*P* value	Adjusted odds ratio	95% CI
All encounters (N=32,735), n (%)	949 (2.9)	1997 (6.1)	<.001	1.64	1.02-2.61
Diabetes (N=24,138), n (%)	724 (3)	1472 (6.1)	<.001	1.44	0.73-2.85
Diabetes with HbA_1c_^b^ above individualized goal (N=12,786), n (%)	384 (3)	793 (6.2)	<.001	1.80	0.92-3.52
Diabetes with HbA_1c_≥8% (N=8463), n (%)	288 (3.4)	609 (7.2)	<.001	1.93	1.01-3.67
Hypertension (N=22,127), n (%)	708 (3.2)	1416 (6.4)	<.001	1.36	0.7-2.62
Hypertension with BP^c^ above goal (N=8515), n (%)	315 (3.7)	553 (6.5)	<.001	1.42	0.72-2.79
Suboptimal lipid management (N=17,765), n (%)	533 (3)	1030 (5.8)	<.001	1.41	0.76-2.61
ASCVD^d^ (N=6679), n (%)	274 (4.1)	448 (6.7)	<.001	1.43	0.77-2.63
Tobacco use (N=7449), n (%)	253 (3.4)	559 (7.5)	<.001	2.14	1.31-3.51
BMI≥30 kg/m^2^ (N=19,838), n (%)	575 (2.9)	1230 (6.2)	<.001	1.45	0.75-2.8

^a^Age (continuous), sex (female/male), and race (White/Black/other) were included in multivariable logistic regression models.

^b^HbA_1c_: glycated hemoglobin A_1c_.

^c^BP: blood pressure.

^d^ASCVD: atherosclerotic cardiovascular disease.

## Discussion

### Principal Findings and Implications

SDM has been recommended for years as a strategy to improve outcomes for patients with chronic disease [28]. However, in the context of other technical accomplishments of this decade, the adoption of SDM beyond rather simple process prompts and reminders has been incredibly slow. The reasons for slow uptake are numerous, including the challenges related to developing, maintaining, and updating SDM content; workflow constraints in busy health care settings; and lack of evidence, until recently, directly correlating SDM use with improved patient outcomes [29]. High-quality SDM has become more widely available to improve quality of care and promote evidence-based standards [30]. To avoid influencing patient and clinician behavior and medical decision-making in nonevidence-based ways, it is important that the SDM developers avoid financial conflicts of interest, use of medication brand names, and biases introduced through commercialization strategies. However, adoption and implementation of high-quality SDM can be costly to care systems, and almost no data are available to describe directly how SDM impacts coding and billing factors that can affect revenue generation.

The SDM system implemented for this study was developed at HealthPartners Institute through a series of federally funded research grants by a team with no financial conflicts of interest and with the main objective of improving patient outcomes. Previous versions of the SDM system had been proven to improve patient outcomes for targeted individuals with diabetes and several cardiometabolic conditions as well as SMI in RCTs [4,5,15,31]. It required minimal staff training for implementation. It has been integrated into external care systems through business associate agreements, service agreements, and assurance of secure data transfer between EHRs and the SDM web service. It currently requires about 4 to 6 months of commitment by the recipient organization to conduct data mapping, programming, and testing prior to implementation, although this work may be streamlined in the future with increasing EHR data interoperability and improved FHIR applications. This SDM is currently in use at all HealthPartners and Park Nicollet primary care clinic systems in Minnesota and Wisconsin and external care organizations in rural Minnesota and 10 other states through collaborative research agreements, with over 250,000 web service calls per month. Because SDM algorithms are maintained and the SDM system output is delivered through web-based functionality, there are no dissemination-related geographic limits or boundaries to overcome. When research projects have ended, the annual costs for keeping the SDM system clinically up to date for diabetes and cardiovascular conditions and maintaining required informatics technology have been modest (estimated at US $200,000 annually) and have been shared by participating care systems. However, even for a clinically successful SDM technology such as this one, dissemination and scalability efforts have been hampered by the inability to demonstrate the value proposition to care delivery systems, with the typical entities deciding whether to adopt the SDM and pay for its integration and maintenance [1].

Although this analysis was exploratory in nature, it was rigorously conducted, and the findings were quite consistent across all coding variables assessed. The findings were compatible with what would be expected if the SDM increased clinician time and attention to important care gaps identified by the SDM. The increase in the appropriate CPT billing codes observed in SDM-targeted encounters is important to care delivery models that rely on FFS reimbursement to capture the increased time and level of medical decision-making at these encounters. Without this appropriate alignment of billing codes, provision of additional value in care may go inadequately incentivized due to reduced FFS reimbursement [32]. Providers tend to systematically underestimate the value of their medical decision-making, which can lead to reduced revenue to support high-quality care [11]. To the extent that using this SDM system increased the amount of time, number of clinical issues addressed, and complexity of medical decision-making at encounters, the increased levels of CPT coding observed are clinically justified and may, as in several of our published studies, improve patient health [11].

In many health systems, FFS reimbursement models are transitioning to or being blended with value-based models [32,33] driven by federal programs such as medical homes and accountable care organizations [33,34]. Value-based models are also being adopted by commercial lines of business, with nearly two-thirds of payments now based to some extent on value [35,36]. Many of these models use a risk adjustment factor (RAF) based on patterns of diagnostic coding to determine the amount of payment to appropriately care for patient populations, assigning a higher RAF to the care of more complex patient populations [33,37]. For many health care organizations, attention to diagnostic coding for risk adjustment has become a top priority to improve care and sustain appropriate reimbursement for the populations they serve under value-based agreements. Some care systems are implementing software programs to explicitly promote accurate diagnosis of chronic conditions [38] in conjunction with mechanisms to ensure sufficient clinical documentation of care to support the diagnostic codes [39,40]. This SDM system did not contain features to explicitly encourage increased diagnostic coding at visits. The observed increases in CPT coding for chronic conditions addressed by the SDM in intervention clinics were not enough to achieve statistical significance with the limited number of clinics included in the analysis (N=17), but further research is warranted, given the consistency of coding changes.

Previous work has established the clinical benefits of using this SDM system, but fostering the business case for implementation and maintenance is critical to scalability and broad dissemination of SDM technology. For this SDM system, implementation and maintenance costs (excluding research-related costs) are known. A formal cost-effectiveness analysis demonstrated cost-effectiveness with likely cost savings to payers at scale [8], but more research is needed to demonstrate that the use of SDM systems does not negatively impact revenue generation. The data presented here demonstrate for the first time that outpatient SDM use at the point of care for patients with DM, SMI, and high CV risk increases high-complexity CPT level of service codes. It accomplishes this by broadening the clinical content of the visit while guiding clinician and patient attention to specific evidence-based clinical actions with potential substantive benefits to a particular patient at the time of a clinical encounter.

These effects of the SDM system could improve short-term revenue generation for a care delivery system. For example, under the assumption that scheduling systems did not change and physician productivity was not affected, the estimated magnitude of revenue generation based on these CPT data for targeted encounters (34,300 SDM-eligible visits) would be approximately US $63,919 over 12 months in a model that assumed that all encounters received published Medicare FFS CPT reimbursement rates (published 2018 revenue rates for CPT codes 99212, 99213, 99214, and 99215 are US $45, 74, 109, and 148, respectively) [13]. In a pure value-based reimbursement model or a blended model with FFS, any positive impacts on diagnostic coding and quality of care would also be expected to increase revenue. As the clinical scope of SDM technology expands beyond CV domains, the extent of the SDM impact on a higher proportion of primary care visits could further enhance the SDM business case.

A number of factors limit the interpretation of the data we present here. First, the data were derived from a single care delivery system and should be replicated elsewhere and in larger studies. The SDM system we evaluated is now being used in many other care systems, which would enable such additional analyses. However, other SDM developers should consider assessing revenue impacts and the impact on physician productivity, such as the production of relative value units, which may vary according to payment models. Second, the value of the increased revenue is only justified by improved quality of clinical care delivered and improved clinical outcomes. Any increases in revenue from billing and coding changes due to the intervention would be in addition to what could be expected through higher incentive payments from better quality outcomes. We have shown in published clinical randomized trials that this SDM system improves clinical outcomes, but future investigations should jointly consider the clinical and economic impacts of SDM technology from the point of view of both the payer and the care delivery system. Third, we focused on encounters made by patients who had the potential for diabetes improvement or CV risk reduction at their clinic visit. It remains to be seen if similar findings would apply to other chronic disease conditions or different acute or preventive care needs. Our results relate to CPT coding practices in a care delivery system that provides close oversight and routine audits of clinician coding practices to ensure accuracy and avoid fraud. Coding practices, and thus the impact of SDM systems on coding practices, may vary across care delivery organizations. The data used to for this analysis were collected before the 2021 changes to the Center for Medicare and Medicaid Services (CMS) Physician Fee Schedule to “reflect a broader administration-wide strategy to create a health care system that results in better accessibility, quality, affordability, empowerment, and innovation” [41]. Further studies are needed to assess the SDM impact with these newest CMS changes that attempt to simplify billing and coding for office-based services and compensate physicians for additional time spent with patients. Lastly, there are SDM-related factors that could positively influence revenue generation in any of these models, but they must be considered in the context of implementation and maintenance costs of SDM systems and the costs of promoting treatments that may be more intensive.

### Conclusions

This analysis demonstrates that use of an SDM system with proven clinical effectiveness was associated with significantly higher levels of CPT level of service 5 coding and with consistent but nonsignificant increases in ICD-10 coding at routine primary care encounters of patients with diabetes and uncontrolled cardiometabolic conditions. An appropriate shift in CPT coding was observed with a significantly increased proportion of encounters coded as high complexity for patients with poorly controlled diabetes and tobacco dependence. The study provides novel and important information that may inform the business decisions related to implementation of SDM technology to improve quality of care for targeted conditions.

## Appendix

Multimedia Appendix 1 CONSORT-eHEALTH checklist (V 1.6.1).

## Abbreviations

BPA: best practice advisory
BP: blood pressure
CDS: clinical decision support
CMS: Center for Medicare and Medicaid Services
CPT: current procedural terminology
CV: cardiovascular
DM: diabetes mellitus
EHR: electronic health record
FFS: fee for service
FHIR: Fast Healthcare Interoperability Resources
HbA_1c_: glycated hemoglobin
ICD-10: 10th Revision of the International Classification of Diseases
MUMPS: Massachusetts General Hospital Utility Programming System
RAF: risk adjustment factor
RCT: randomized controlled trial
SDM: shared decision-making
SMI: serious mental illness

## Data Availability

The data sets used and analyzed in this study are available from the corresponding author on reasonable request.
